# Cardiac function and autonomic cardiac function during a multi-stage cycling event: a brief report

**DOI:** 10.3389/fspor.2024.1356577

**Published:** 2024-07-29

**Authors:** Vincent Menard, Anna Barrero, Thibault Lachard, Lucien Robinault, Lingxia Li, Frederic Schnell, François Carré, Solène Le Douairon Lahaye

**Affiliations:** ^1^ M2S Laboratory, University of Rennes 2, Rennes, France; ^2^ Department of Sports Medicine, University Hospital of Rennes, Rennes, France; ^3^ Independent Researcher, Auckland, New Zealand; ^4^ Center of Clinical Investigation of Rennes, CIC-CIT INSERM 1414, Rennes, France; ^5^ INSERM, LTSI-UMR1099, University of Rennes 1, Rennes, France

**Keywords:** endurance, exercise-induced fatigue, heart rate variability, echocardiography, athlete

## Abstract

**Introduction:**

Prolonged and repeated exercise performed during an ultra-endurance event can induce general and cardiac fatigue known as exercise-induced cardiac fatigue. Our objective was to find a possible correlation between the cardiac function and the autonomic cardiac function.

**Methods:**

During a multistage ultra-endurance event, a female well-trained cyclist underwent daily rest echocardiography and heart rate variability measurements to assess the cardiac function and the cardiac autonomic function.

**Results:**

The athlete completed 3,345 km at 65% of her maximum heart rate and 39% of her maximum aerobic power. A progressive improvement of the systolic function for both the left ventricle and the right ventricle was observed during the event.

**Discussion:**

Alterations were observed on the cardiac autonomic function with an imbalance between sympathetic and parasympathetic, but there was no sign of a significant correlation between the cardiac function and the autonomic cardiac function and no signs of cardiac fatigue either. Further analysis should be performed on a larger sample to confirm the obtained results.

## 1 Introduction

Due to a high cardiac workload, prolonged intense exercise, such as that performed during ultra-endurance events, results in an exercise-induced cardiac fatigue (EICF) (1). This phenomenon is characterized by transient cardiac function alterations associated with an increase in the cardiac damage biomarkers after athletes perform ultra-endurance events, whether isolated (2) or repeated over several consecutive days (3).

Although the association is not clearly demonstrated, the repetition over time of an EICF episode could have a negative impact on the cardiovascular health of athletes (4, 5).

To date, EICF is generally evaluated through conventional echocardiography, tissue Doppler imaging, speckle tracking techniques, or cardiac biomarkers (6–8). Although they are relevant, these evaluation methods are not easy to use in the routine of athletes.

The cardiovascular control by the autonomic nervous system (ANS) can be indirectly assessed through the heart rate variability (HRV) (9–11). The HRV assessment is known to be a reliable, affordable, and non-invasive tool for assessing the autonomic cardiac function and for evaluating the physiological state (fitness vs. fatigue) of athletes (12).

Thus, the objective of this study was to describe the evolution of the cardiac function assessed by echocardiography and the autonomic cardiac function assessed by the HRV during an ultra-endurance event in a well-trained female cyclist, thereby determining their relationship.

## 2 Method

As in our previously published studies (13, 14), this scientific project was carried out as part of the sports project “Donnons des elles au vélo J-1,” which aimed to promote women's cycling.

### 2.1 Participant

One athlete was included in this case report. She received daily HRV measurements and was subsequently subjected to an echocardiography examination. The participant was a well-trained (maximal aerobic power output: 325 W and $\dot{V}O_{2}$ max: 62 ml.min.kg^−1^) Spanish female cyclist (32 years old, 53 kg, 169 cm).

### 2.2 Description of the cycling event

The cyclist performed the ultra-endurance events from the Tour de France using the men's routes. Details about the event have already been published (13, 14).

To preserve and respect the full rest of the athlete in the morning, no data were recorded on the 2 days of rest during the event between the 9th and 10th stages and between the 15th and 16th days.

### 2.3 Load analysis

During the different stages, the rate of perceived exertion (RPE) was assessed using the Borg CR-10 Scale for subjective load (15). The mean heart rate (HR) during the event were compared to the maximum theoretical HR of the athlete, as was done for the mean normalized aerobic power (NP) output, to also assess the exercise load of the event.

### 2.4 Echocardiography for the cardiac function evaluation

The echocardiography analysis protocol used was previously described (16).

**Figure 1:**
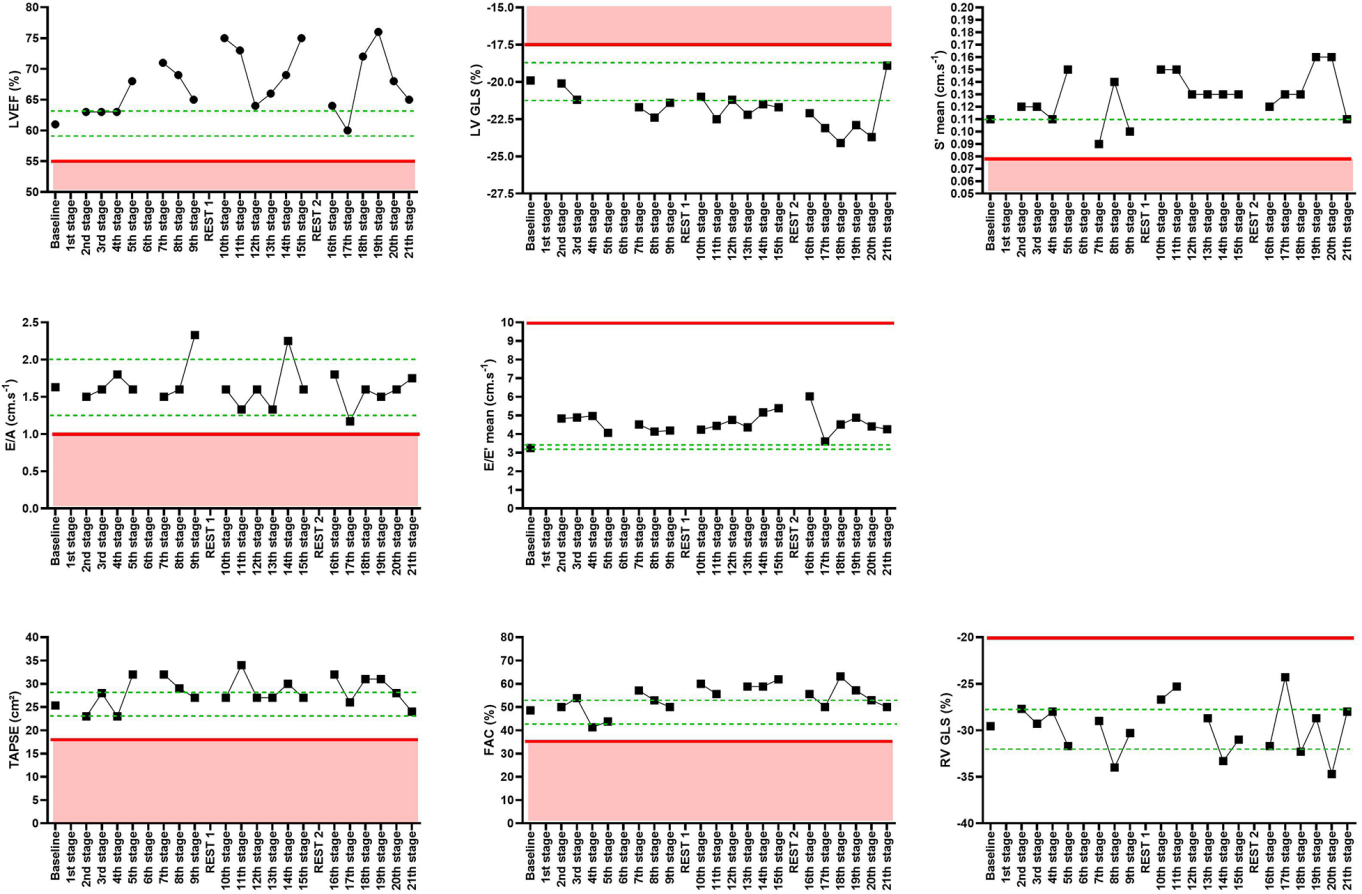
Echocardiographic parameters in a graphic presentation with the minimal and maximal ranges for the baseline and the clinical threshold for the left ventricle ejection fraction (LVEF), left ventricle global longitudinal strain (LV GLS), mean S′ wave, E/A ratio, E/E′ mean ratio, tricuspid annular plane systolic excursion (TAPSE), fractional area change (FAC), and right ventricle global longitudinal strain (RV GLS) min /max for the rest days - - - -; clinical acceptable value ———.

The participant underwent resting echocardiography using Vivid Q (GE Vingmed Ultrasound AS, Horten, Norway) for 3 days before the event to assess the possible variations of each parameter during days without physical activity, and then daily in the morning just after waking up, except on the two rest days. All the variations were compared to the baseline (Figure 1).

All echocardiography examinations were performed, and the results were read and interpreted by a single cardiologist blinded to the clinical data as to prevent any inter-operator variability.

### 2.5 RR interval recording and HRV analysis for autonomic cardiac function evaluation

The RR interval recording and HRV analysis protocols used were previously described (13).

The baseline pre-event RR intervals were collected daily for 3 days before the event to obtain a basal HRV state. All resting recordings were made in a fasted state, right after awakening and before the cyclist gets up, and thus before the echocardiography.

Both time and frequency domain HRV analyses were performed.

### 2.6 Statistical analysis

All the statistical analyses were performed with Python software version 3.9.1.2 (Python Software Foundation, USA). The Pearson product moment correlation was used to evaluate the correlations between the echocardiography and HRV parameters.

The significant threshold was set at *p *< 0.05, and the correlation threshold was set at *r *> 0.8/−0.8 to only keep the strong correlations.

## 3 Results

For the HRV analysis, out of the 21 stages, 20 stages had usable data for the supine position, and 18 had usable data for the standing one. For the echocardiography analysis, 19 stages were usable.

### 3.1 Load analysis

The athlete performed 3,345 km in 21 stages. During the event, the mean heart rate was 65% of her maximum HR; the mean NP output was 39% of her maximum aerobic power; and, finally, the mean RPE was a 5 on the Borg CR-10 Scale.

### 3.2 Evolution of the cardiac function: echocardiography parameters

The echocardiographic data before and during the event are shown in Figure 1.

All echocardiographic data parameters were compared to the baseline, with a range of minimum and maximum for each parameter. The evolution of each parameter was also compared to the clinical norms presented in Figure 1.

#### 3.2.1 Left ventricle systolic function

All along the 21 stages, an increase in the LV systolic function was observed. For the left ventricle ejection fraction (LVEF), this trend was evident, despite the decrease of the systolic function in some of the stages. The LVEF stayed under clinical norms during the whole event. The same trend was observed for the left ventricle global longitudinal strain, which increased from −19.9% during baseline to −23.7% during the 20th stage. An improvement of the S′ mean wave was also reported (baseline: 0.11 vs. 20th stage: 0.16). A decrease was observed on the 7th stage and the 9th stage, but the S′ mean remained under the pathological threshold.

#### 3.2.2 Left ventricle diastolic function

There were variations for the E and A waves and for the E/A ratio, although the E/A values remained close to the baseline. These variations always stayed above the clinical norms of the pathological threshold (<1). An upward trend was also observed for the E/E′ mean, which always stayed above the baseline values.

#### 3.2.3 Right ventricle systolic function

For the RV fractional area change, tricuspid annular plane systolic excursion, and global longitudinal strain, an upward trend was reported during the 21 stages of the event. No signs of significant clinical degradations were found.

### 3.3 Evolution of the autonomic cardiac function: HRV parameters

#### 3.3.1 Supine HRV parameters

**Figure 2:**
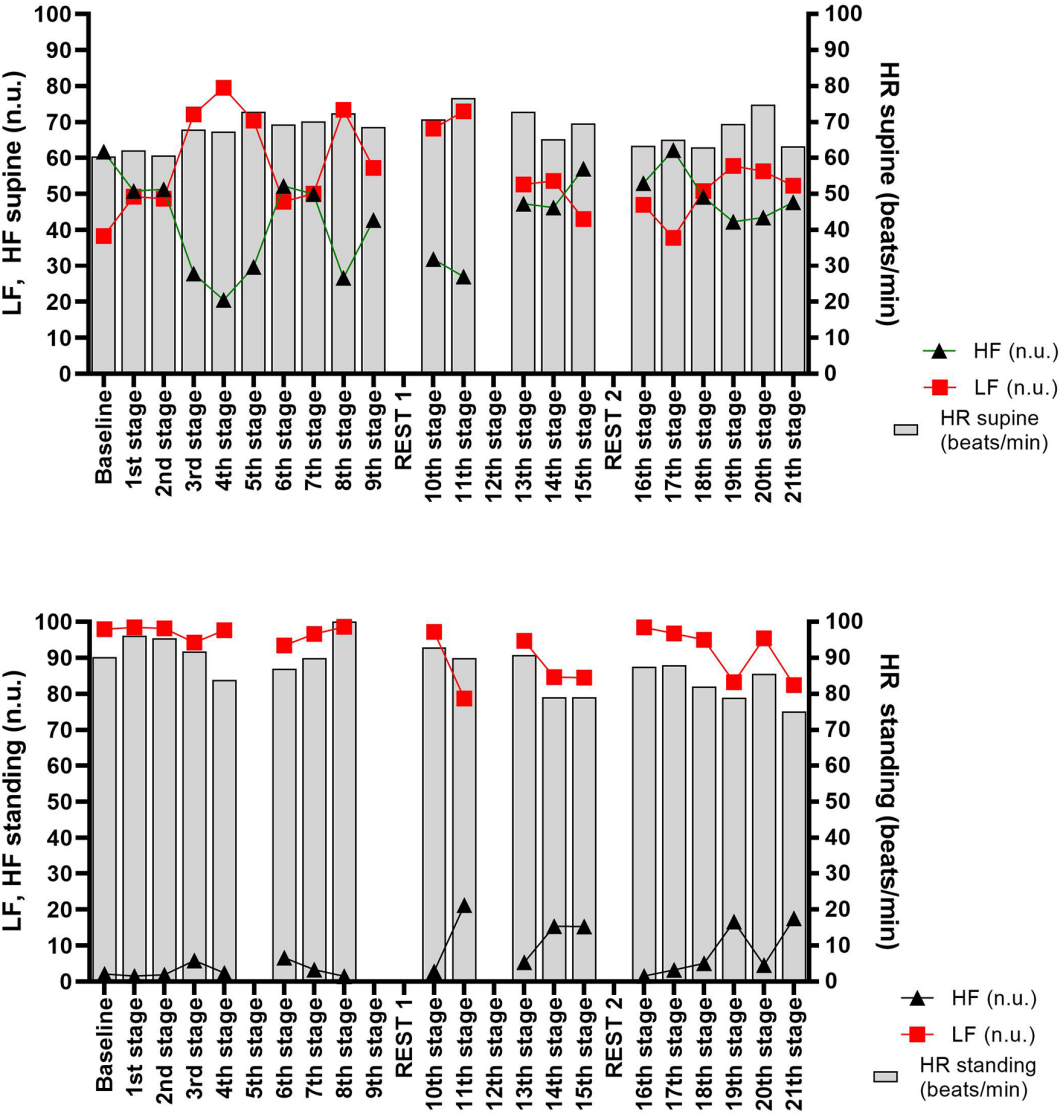
HRV parameters in the supine and standing positions in a graphic presentation with heart rate (HR), low frequencies (LF), and high frequencies (HF) in normalized units (nu).

The supine HRV data before and during the event are shown in Figure 2.

The supine HR tended to increase all along the event, starting from the morning of the 3rd stage. This was associated with an increase of the LF and a decrease of the HF (Figure 2). The LF and HF evolutions induced an inversion of the supine autonomic balance (3rd-stage morning). This imbalance was more or less important all along the event, function of the length, and difficulty of the stages. Indeed, the increase of the HF and the LF and the decrease of the HF were linked to the increase in the RPE on these stages.

#### 3.3.2 Standing HRV parameters

The standing HRV data before and during the event are shown in Figure 2. A trend to standing HR decrease was observed all along the event. This was associated with the decrease of the LF and the increase of the HF, particularly after the 10th stage (Figure 2).

After the investigation of the lnRMSSD and the lncRMSSD (17, 18), no conclusive trends were found in relation to their respective evolutions through the different stages of the athlete's performance.

### 3.4 Correlation between the cardiac function and autonomic cardiac function parameters

No correlation was observed between the cardiac function and the autonomic cardiac function. Indeed, no matter the echocardiographic or the HRV parameter chosen, no significant correlation was found.

## 4 Discussion

The objective of this study was to evaluate the relationship between the evolutions of the cardiac function and the autonomic cardiac function during an ultra-endurance multi-stage event.

The main result of our case study is that no significant correlation is found between the cardiac function and the autonomic cardiac function, which were assessed by echocardiography and HRV analysis, respectively.

While the cardiac function seems to be improved during the 21 stages of the ultra-endurance event, the autonomic cardiac function is more likely to be disrupted all throughout the event.

Indeed, no clinical signs of cardiac function degradation or fatigue were observed either for the systolic and diastolic functions of the LV and the systolic function of the RV. It even seems to improve during the ultra-endurance event.

By contrast, we observed changes in the resting autonomic balance (HRV supine measure) starting from the third day. Indeed, the HRV parameter analysis revealed a decrease of the parasympathetic influence (HF) and an increase of the sympathetic one (LF), which led to an increase of the resting HR. These changes remained during the duration of the event, with a modification of the autonomic imbalance between the sympathetic and parasympathetic influences that could indicate a potential fatigue, regardless of the duration and the difficulty of each stage.

Concerning the autonomic response to orthostatic stress (HRV standing measure), we observed a decreased LF, followed by an increased HF, which induced a decreased HR. It seems possible that the stress induced by the repetition of the stages adds up without sufficient time for the athlete to recover.

The physiological adaptations required by the ultra-endurance multi-stage event were high enough to induce several variations in the cardiac autonomic function, which was assessed via the HRV analysis. However, these physiological adaptations were not significant enough to alter the cardiac function, which was assessed by echocardiography.

The study presents some limitations. For logistical reasons, we did not take into account the variations in hydration, body weight, and blood pressure that may be induced by this type of ultra-endurance exercise. However, we cannot deny the importance of these parameters in the evolution of the cardiac function due to their impact on the pre- and afterload. Nevertheless, in other studies conducted in our team, no significant variation in the body weight, systolic and diastolic pressures (16), or LVED (19) was observed after the same type of ultra-endurance events. In this study, the fact that LVED did not decrease, that the athlete could hydrate without restriction during exercise, and that all measurements were performed after a night of sleep are in disfavor of a possible dehydration.

## 5 Conclusion

Our results suggest that an ultra-endurance event at moderate intensity can affect the autonomic cardiac function without any sign of cardiac fatigue, as assessed by echocardiography. These results need to be confirmed in a larger population of athletes.

## Data Availability

The original contributions presented in the study are included in the article/Supplementary Material, further inquiries can be directed to the corresponding author.
