# Corrigendum: Cardiac function and autonomic cardiac function during a multi-stage cycling event: a brief report

**DOI:** 10.3389/fspor.2024.1494555

**Published:** 2024-09-20

**Authors:** Vincent Menard, Anna Barrero, Thibault Lachard, Lucien Robinault, Lingxia Li, Frederic Schnell, François Carré, Solène Le Douairon Lahaye

**Affiliations:** ^1^ M2S Laboratory, University of Rennes 2, Rennes, France; ^2^ Department of Sports Medicine, University Hospital of Rennes, Rennes, France; ^3^ Independent Researcher, Auckland, New Zealand; ^4^ Center of Clinical Investigation of Rennes, CIC-CIT INSERM 1414, Rennes, France; ^5^ INSERM, LTSI-UMR1099, University of Rennes 1, Rennes, France

**Keywords:** endurance, exercise-induced fatigue, heart rate variability, echocardiography, athlete

A Corrigendum on Cardiac function and autonomic cardiac function during a multi-stage cycling event: a brief report By Menard V, Barrero A, Lachard T, Robinault L, Li L, Schnell F, Carré F and Le Douairon Lahaye S (2024). Front. Sports Act. Living 6. doi: 10.3389/fspor.2024.1356577

Incorrect Affiliation

In the published article, there was an error regarding the affiliations for Robinault Lucien. Instead of “*LAMIH, UMR CNRS 8201, University of Polytechnique of Hauts de France, Valenciennes, France*” and “*Center of Chiropractic Research, New Zealand College of Chiropractic, Auckland, New Zealand*”, it should be “Independent Researcher, Auckland, New Zealand”.

The authors apologize for this error and state that this does not change the scientific conclusions of the article in any way. The original article has been updated.

